# The relationship of inclusive climate and peers' attitude on children with disabilities in China: A mediating role of empathy

**DOI:** 10.3389/fpsyg.2022.1034232

**Published:** 2022-12-14

**Authors:** Wangqian Fu, Yawen Xiao, Cui Yin, Tianqi Zhou

**Affiliations:** ^1^School of Special Education, Beijing Normal University, Beijing, China; ^2^School of Education, Beijing Sport University, Beijing, China; ^3^Faculty of Education, Beijing Normal University, Beijing, China

**Keywords:** attitude to peers with disabilities, inclusive climate, empathy, China, school climate

## Abstract

**Objective:**

Peer attitude towards children with disabilities can significantly impact on the successful implementation of inclusive education. The current study examined the attitude of Chinese students toward peers with disabilities in relation to inclusive climate and empathy.

**Method:**

A total of 392 typically developing students (aging from 10 to 17 years old, with average age of 12.7) in primary and middle schools participated in the study by reporting the Inclusive Climate Scale, Peers Attitude to Students with Disabilities Scale, and Balanced Emotional Empathy Scale.

**Results:**

We found that students held a neutral attitude to peers with disabilities, and girls were more acceptable than boys. There were positive relationships among inclusive climate, empathy and attitude to classmates with disabilities. The mediation analysis showed that empathy played a partial mediation role between inclusive climate and the peers' attitude towards children with disabilities.

**Conclusion:**

Implications for improving students' attitude on peers with disabilities were discussed in the paper.

## Introduction

Inclusive education is a worldwide trend in the education of children with disabilities. The government in China makes efforts to promote the inclusion of children with disabilities as well. From 2012 to 2019, the number of students with disabilities attending ordinary schools increased from 199,800 to 394,300, with an increased rate of 97.3% (Moe, 2020). Attitude of classmates have been considered one of the major problems in inclusive education (Çiçek Gümüş and Öncel, 2022), negatively influencing the participation of students with a disability in school contexts (Maggio et al., 2021). Research shows that peer relationships are an important context for developing children's socialization and are a source of social support and security in order to meet children's social needs (Zou, 1998; Aparicio Puerta and Polo Sánchez, 2022). Peer attitude toward children with disabilities can significantly impact their self-confidence and, hence, their social acceptance. The degree to which children are liked or accepted by their peer group plays a decisive role in the healthy personality development of children with disabilities (Sang, 2003; Yang et al., 2012).

Students with disabilities are more sensitive and vulnerable due to their special characteristics and are more likely to be rejected by their peers (Ostrosky et al., 2015; Nota et al., 2018). For instance, students with disabilities have greater levels of interpersonal conflict at school, loneliness, and isolation (Hogan et al., 2000). It was found that for children with autism spectrum disorder, their peer relationships were not promising. They were less accepted in their interactions with peers, had fewer and lower quality friendships, were more isolated, had less social network centrality, and had difficulty integrating into the classroom social network than a typically developed child (Kasari et al., 2012; Locke et al., 2012; Zhang et al., 2019). Even a small group of students holding negative attitude can make the school life of children with a disability very difficult (McDougall et al., 2004). In an inclusive education setting, peer attitude has a significant impact on students with disabilities. The attitude of the general student population toward students with disabilities will be directly related to the ability of students with disabilities to adapt to school life, acquire social values and competencies, and achieve healthy cognitive and personality development (Zou, 1997). On the one hand, negative attitude make students with disabilities more vulnerable to physical and psychological bullying at this stage. On the other hand, the psychologically damaged in peer relationships may contribute to character deficits in the current and future individual development of children with disabilities.

Apart from the influence on children with disabilities, the peers' attitude toward inclusive education is also associated with typically developed children's development. Peers who befriend students with disabilities hold more positive attitude, and these peers might develop a greater understanding of and more sensitivity toward such students and perceive them more positively (De Boer et al., 2012a). Peers holding positive attitude toward both their peers with disabilities and inclusion can also benefit themselves from the experience of being in an inclusive school environment (Peck et al., 1990; Helmstetter et al., 1994; Kishi and Meyer, 1994; York and Tundidor, 1995; Fisher et al., 1998; Fisher, 1999; He and Zuo, 2012). General students may generate a more positive attitude toward difficulties through their engagement with students with disabilities.

The process of information disclosure and mutual understanding, typified by friendship relationships, triggers positive feelings that can be transferred from the individual concerned to his or her entire social group (Pettigrew and Tropp, 2008). Intimacy has been shown to be an indicator of friendship quality: the higher the level of intimacy, the more friends are treated as part of themselves (Davies et al., 2013). The positive atmosphere created among students in an inclusive educational environment leads to cross-group friendships and changes the negative attitude of ordinary students. Despite the importance of peers' attitude to children with disabilities for the development of both children with disabilities and themselves, the research in China mainly focuses on the attitude of teachers' groups rather than peers' attitude. Much less about examining the influencing factors on peers' attitude on children with disabilities. From a bioecological perspective, individuals' attitude toward inclusive education is not only influenced by contextual factors (e.g., school climate) but also related to personal factors (McDougall et al., 2004; Ginevra et al., 2021), which should be further explored in order to better understand it and take measures to improve it in the Chinese context.

### Inclusive educational climate and peers' attitude of inclusive education

Peers' attitude toward inclusive education is associated with many factors, such as gender, age, frequency of contact with students with disabilities (Chen and Lu, 2006; Maggio et al., 2021), knowledge of disabilities (Lu et al., 2021), and school context (e.g., school climate) (McDougall et al., 2004). School climate in which students live is an important factor in their attitude toward inclusive education. School climate reflects the quality and character of school life, the norms, goals, values, interpersonal relationships, teaching and learning practices, and organizational structure of a school (Cohen et al., 2009). In the context of inclusive education, an inclusive school climate refers to a relatively permanent and stable atmosphere in which teachers and their surroundings interact with each other over a long period of time in order to implement inclusive education (Wang et al., 2020). McDougall et al. (2004) suggest that school climate is important in order to promote student attitude toward peers with disabilities, including equitable school goal structures and interpersonal support for students from other students and teachers.

Positive school climate created in the general school environment, between teachers and students, and among students plays an important role in changing the negative–neutral attitude of the average student toward inclusive education. The effect includes the support and attention to the smooth promotion of inclusive education under the leadership of the principal, the implicit influence of the campus culture, and institutional protection at the school level. The Chinese government makes effort to promote inclusive climate in general schools, including organizing the training of all principals and teachers in general schools on inclusive education, emphasizing a barrier-free environment (Central People's Government of China, 2021).

### Relationship between empathy and peers' attitude toward inclusive education

Empathy is an emotional response based on an individual's understanding of another person's emotional state or condition, which is equal to or similar to the feelings being experienced or likely to be experienced by another person (Eisenberg et al., 2006; Segal, 2018). Empathy can help individuals move away from self-centeredness, build peer relationships, develop prosocial behavior and emotion regulation, and is the basis for the development of social competence (Guan, 2018), which is significant in order to form and maintain different forms of social relationships (Robert et al., 2014). Cohen et al. (2009) argue that in social interactions, individuals need to refer to the mental states of others and cannot understand their intentions and respond appropriately without taking into account verbal and non-verbal factors. In the interactions with students with disabilities, the empathy of typically developed students plays a positive role in the development of good attitude toward inclusive education (Vuorinen et al., 2019).

Students' empathy is a multidimensional structure that encompasses both cognitive and emotional responses (Boele et al., 2019). Emotional empathy is when individuals empathize and have alternative experiences to the emotions of others, and cognitive empathy is when individuals are able to identify and understand the emotions or perspectives of others (Huang and Su, 2012). Empathy has been shown to assist in individuals' abilities to build deeper connections, regulate emotions, and has also been found to promote helping behavior (Griffin, 2019; Loerger et al., 2019). Mirete et al. (2020) find empathic concern can predict good attitude toward people with intellectual disabilities among university students. Peers' attitude toward inclusive education are influenced by their own life experiences and knowledge of disability, which increases the individual's understanding of the emotions and feelings of others, thus, increasing the likelihood that the individual will respond empathetically to others. In addition, individual students belong to their own groups and have multiple group identities. When individuals come into contact with other groups, they try to put themselves in the other group's perspective, understand the reasons for the other group members' emotions and experience the corresponding emotional state, and this is the process of group empathy. Group empathy occurs when the general student group tries to think from the perspective of a disabled student group (He and Xie, 2018). Therefore, typically developed children's empathy is positively related to positive attitude toward inclusive education.

### The mediating effect of empathy

Environment shapes attitude to varying degrees and predisposes them to reject or accept something (Verkuyten and Steenhuis, 2005; Griffiths and Nesdale, 2007). General students can be influenced by a variety of factors in his or her environment, resulting in different attitude toward inclusive education. School climate in which the students live is associated with their attitude toward inclusive education. In cognitive response theory, individuals are active in processing information to produce cognitive responses, rather than just passive receivers of any information of the environment (James and Gutkind, 1985). That is, personal psychological factors (e.g., empathy) may play a mediating effect.

From the point of view of self-determination theory, in a good atmosphere of inclusive education, the basic psychological developmental needs of the average individual student are met based on environmental support (Reeve, 2012; Diseth et al., 2018), after which, the individual develops more prosocial behavior (Gagné, 2003), thus, helping them to develop good attitude toward inclusive education. Moreover, empathy is a key factor in the formation of a good atmosphere between individuals and even groups during the process of contact (Batson and Ahmad, 2010). In a good atmosphere of inclusive education, the two develop empathy through contact with each other. On the one hand, the empathy that ordinary students have for students with disabilities reduces stereotypes and increases positive evaluations of individuals and groups of students with disabilities (Batson et al., 1997). On the other hand, it extends from forming a positive and positive evaluation of an individual to forming a positive evaluation of a group, and based on forming a positive evaluation of students with disabilities, it promotes positive actions for ordinary students, which in turn, helps ordinary students to develop a positive attitude toward inclusive education.

Overall, previous studies have shown that peers' attitude toward inclusive education is very important to the development of students with disabilities in inclusive settings. Furthermore, it is also related to typically developing children's development. Many studies indicate that school climate and empathy bear importantly on peer attitude. However, there are some limitations. First, there has been a significant expansion in the enrollment of students with disabilities learning in regular schools in China, while only a few studies investigated peers' attitude, reporting peers' holding negative attitude (Ning, 2018; Zhang et al., 2018). In the trend of comprehensively promoting inclusive education for children with disabilities in China (Central People's Government of China et al., 2017), it is important to be able to understand the attitude of students without disabilities toward the inclusion of students with disabilities. Some of the obstacles experienced by students with disabilities are associated with how best they can be accepted by their peers (Kapinga, 2020). Second, many studies examined one or two attitude components for which different questionnaires were used (De Boer et al., 2012b). Vignes et al. (2009) recommend an instrument in which all three components are included, as findings may vary according to the type of component assessed. In addition, although there is a vast literature on the topic of peers' attitude toward inclusive education worldwide, most studies tried to investigate the connection separately focusing on either school climate or empathy contributing to peers' attitude (Batson and Ahmad, 2010) and the systematic empirical research to considering the relationship of school climate and empathy in the influence of peers' attitude is few.

To address these gaps, we examined peers' attitude toward children with disabilities in China. Successful implementation of inclusive education in developing countries such as China is not an easy venture; therefore, it is important to achieve a comprehensive understanding. It can contribute to the multicultural finding on the topic as well. Furthermore, we explored the relationship between school climate, empathy, and peers' attitude in order to suggest steps that may be undertaken to ameliorate this problem by mediation hypotheses. According to Shadish (1996), it is useful to utilize the mediation hypotheses in order to develop and advance the theories of human behavior. In addition, they can contribute to the practitioner having an insight into (a) the process and the reason relationships unfold and (b) where in the behavior people having problems, practical interventions need to be taken.

## Methods

### Participants and procedure

The data were collected from six primary and secondary schools implementing inclusive education in Beijing, which meant that students with disabilities were enrolled in those schools. We contacted the principals of the elementary schools to get permission to deliver the questionnaires to their schools. The volunteer principals helped to obtain the consent of students and their parents. Considering the reading ability of completing the paper questionnaires by students, the research subjects were students above the fifth grade of primary school as well as middle school, who were between the ages 10 and 17 (average age = 12.7). The demographic information is shown in Table 1.

**Table 1:** Demographic information of participants.

		***N***	**%**
Gender	Boys	200	51.02
	Girls	192	48.98
Age	10–12	89	22.7
	13–15	285	72.7
	16–17	18	4.6
Only-child in family	Only one	196	50
	One above	196	50
Grade	5–6	71	18.11
	7–9	321	82.89
School level	Primary school	71	18.11
	Secondly school	321	81.89

It is widely understood that the use of larger samples in applications of factor analysis and mediation analysis tends to provide results such that sample factor loadings are more precise estimates of population loadings and are also more stable, or less variable, across repeated sampling. According to Cattell (1978), the ratio of sample size and the number of variable items should be in the range of 3–6. To meet the criterion, the sample size should be more than 320 (with 10% invalid questionnaires in assumption) in the study. A total of 400 questionnaires were delivered to the six schools and eight questionnaires were checked for double choice and incompleteness, resulting in 392 valid questionnaires. The sample consisted of 48.98% girls and 51.02% boys. Of those, 18.11% were students in the fifth year of primary school, and 81.89% were students in the fifth year of middle school.

In the basic information of the questionnaire, we investigated the category of disabilities for students with disabilities met by typically developing peers. Although inclusive education has been implemented for decades in China, there has been no specific policy for general schools to create an inclusive environment; about 45.2% of students in our study reported that they did not know the category of disabilities. That was the basic knowledge about disability was not taught to the typically developing students even in the schools enrolled the children with disabilities. Thus, we did not include the variable of the category of disabilities for students in our study.

### Measurement

#### Inclusive climate

The inclusive climate scale (ICS) designed by Schwab et al. (2018) was used in this study. We translated it to the Chinese version and asked two professors in special education to check the expression. The scale consists of 28 items and covers two main components. One is teachers' support and care (e.g., “My teacher cares about all students,” “My teacher makes sure all students are actively involved in most school and classroom activities”), and the other is emotional experience (e.g., “I like coming to school every day,” “I am happy to be at this school”). The ICS uses a five-point Likert scale ranging from 1 (not at all correct) to 5 (completely correct). The total score of 28 questions was used as the student's score after reverse coding some of the questions for scoring.

#### Peers' attitude to students with disabilities

The peers' attitude to students with disabilities scale (PASD) was developed by Rosenbaum et al. (1986) to measure children's attitude toward their peers with disabilities. In its original form, the PASD contained 36 items covering an affective component (e.g., “I am happy to have a child with a disability as a special friend”), a behavioral component (e.g., “I will invite a child with a disability to my birthday party”), and a cognitive component (e.g., “The child with a disability feels sorry for himself”). In Rosenbaum et al.'s (1986) development, it was found that nine of these items did not address the above three components and were deleted. For this study, 27 items were used, with the positively stated and negatively stated items randomized. The PASD was a five-point scale ranging from 1 (strongly disagree) to 5 (strongly agree). Negatively stated items were reverse coded so that the higher scores represented more positive attitude and the total score of the 27 questions was used as the student score.

#### Empathy

The present study used the Balanced Emotional Empathy Scale (BEES) (Mehrabian, 1997) to assess the subjects' levels of empathy. Mehrabian and Epstein (1972) originally developed the Emotional Empathy Tendency Scale (EETS) and continued to update and refine the scale over the next two decades until 1996 when the BEES was finally devised (Mehrabian, 1997). The scale consists of 30 questions, including 15 positively stated questions (e.g., “I am moved when I see a stranger trying to live,” “I am distressed when I see a young person in a wheelchair”) and 15 negatively stated questions (e.g., “A crying child does not get my attention,” “I do not get too involved in my friends' difficulties”). Subjects responded to these prompts using a nine-point Likert format ranging from−4 (for “strongly disagree”) to +4 (for “strongly agree”) (Mehrabian, 1997). In this study, the nine-point Likert format was replaced with a five-point Likert format ranging from 1 (for “not at all true”) to 5 (for “completely true”) in order to facilitate a scoring study. The total score for the negative statements was subtracted from the total score for the positive statements in order to obtain the total scale score.

### Data analysis

In the analysis, the valid sample size was 392. Inclusive climate, empathy, and attitude toward peers with disabilities are observed variables. The standard score of each dimension was taken as the value of each observation variable. To test whether empathy plays a mediating role in inclusive climate and attitude toward peers with disabilities, the mediation model was applied using the PROCESS SPSS 20.0 computing tool.

## Results

### Descriptive statistics

The reliability of the questionnaire was calculated using Cronbach's alpha coefficient. Confirmatory factor analysis was conducted to test the item factor loading. The reliability and factor loading range of each questionnaire are shown in Table 2.

**Table 2:** Questionnaire reliability and factor loading.

**Variable**	**Cronbach's alpha**	**Factor loading**
Inclusive climate	0.917	0.602–0.794
Empathy	0.711	
Attitude to peers with disabilities	0.778	0.399-0.764

The study investigated the inclusive climate, empathy, and attitude toward peers with disabilities of typically developing students in China. Descriptive variables are shown in Table 2. As shown in Table 3, the typically developing students in China perceived a high level of inclusive climate in schools and held a medium level of empathy and attitude toward peers with disabilities. Specially, in the sub-dimension of attitude toward peers with disabilities, they scored highest in the dimension of behavior, with a mean of 3.53. The following sub-dimensions were affection and cognitive in order.

**Table 3:** Descriptive data of variables.

		**M**	**SD**	***t***	**sig**
Inclusive climate	Male	4.234	0.611		
	Female	4.249	0.699		
	Total	4.241	0.655	0.24	0.82
Empathy	Male	3.386	0.435		
	Female	3.573	0.421		
	Total	3.478	0.438	4.31	0.000^***^
Attitude	Male	3.320	0.478		
	Female	3.445	0.494		
Sub-affection		3.33	0.55	1.49	0.137
Sub-behavior		3.53	0.63	3.14	0.002
Sub-cognitive		3.29	0.62	1.47	0.144
	Total	3.382	0.489	2.52	0.012

*** p < 0.001.

### Correlations among inclusive climate, empathy, and attitude toward peers with disabilities

The study also investigated the relationship among all variables. To this aim, Pearson correlation was conducted on inclusive climate, empathy, and attitude toward peers with disabilities. Correlative statistics are shown in Table 4.

**Table 4:** Correlations among inclusive climate, empathy and attitude to peers with disabilities.

	**Inclusive climate**	**Empathy**	**Attitude**
Inclusive climate	1	0.360^**^	0.428^**^
Empathy	0.360^**^	1	0.328^**^
Attitude	0.428^**^	0.328^**^	1

** p < 0.01.

As the earlier table indicates, there was a positive correlation coefficient among inclusive climate, empathy, and attitude toward peers with disabilities. Inclusive climate was significantly associated with attitude toward peers with disabilities of typically developing students (*r* = 0.43, *p* < 0.01) and empathy (*r* = 0.36, *p* < 0.01). Meanwhile, empathy was significantly associated with attitude (*r* = 0.33, *p* < 0.01). Therefore, it can be inferred that the higher the inclusive climate or empathy, the better attitude of typically developing children toward peers with disabilities.

### The mediation effect of empathy

**Figure 1:**
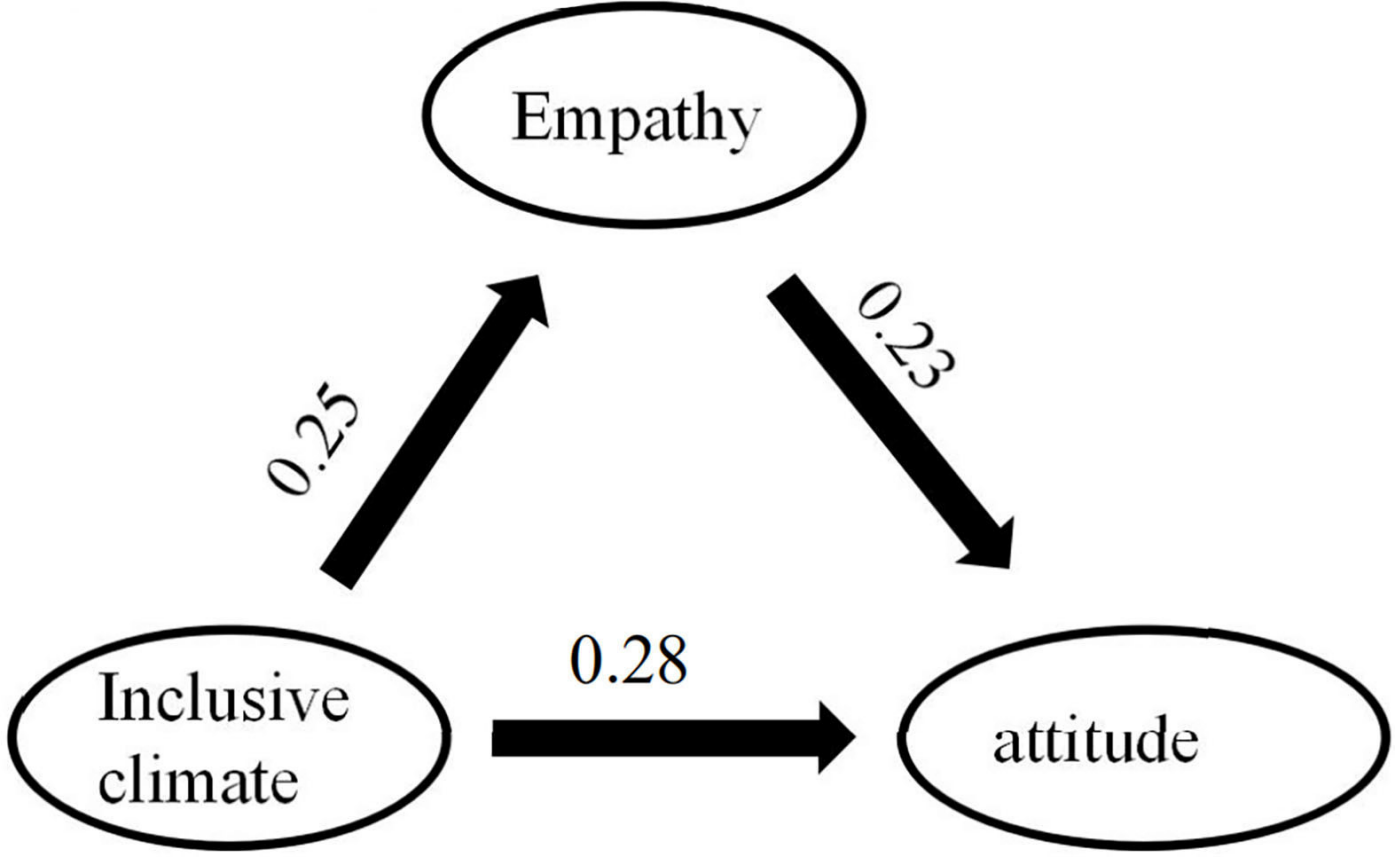
Mediation effect of empathy between inclusive climate and attitude.

Next, the structural equation model (SEM) was conducted to explore the role of inclusive climate and empathy on attitude toward peers with disabilities. The model shows that empathy mediates the influence of inclusive climate on attitude toward peers with disabilities (see Figure 1).

The standard score of each dimension was taken as the value of each observation variable. We tested whether empathy played a mediating role in inclusive climate and attitude toward peers with disabilities (see Table 5). The result showed that the model had a direct role in the influence of inclusive climate on attitude toward students with disabilities, and an indirect path from inclusive climate to empathy then from empathy to attitude toward students with disabilities. The total effect of inclusive climate on peers' attitude to children with disabilities was 0.27, the direct effect of them was 0.21, and the mediating effect of empathy was 0.06. And 95% percentile bootstrap confidence intervals = [0.02,0.10], the interval didn't include 0, indicating that the mediating effect was significant.

**Table 5:** Mediation analyses (5,000 bootstraps).

**Independent variable (IV)**	**Dependent variable (DV)**	**Effect of IV on M**		**Effect of M on DV**		**Effect of IV on DV**		**Direct effect**		**Indirect effect**		**Total effect**	
		**Coeff**	**SE**	**Coeff**	**SE**	**Coeff**	**SE**	**Coeff**	**SE**	**Coeff**	**95%CI**	**Coeff**	**SE**
Inclusive climate	Empathy	0.25^***^	0.03	0.23^***^	0.05	0.21^***^	0.05	0.21^***^	0.05	0.06	0.02, 0.10	0.27^***^	0.04

*** p < 0.001.

## Discussion

This study has begun to address the gaps in our knowledge about the peers' attitude toward students with disabilities in inclusive settings in China, and the relationship between school climate, empathy, and peers' attitude.

The results of this study have found that students' attitude toward their peers with disabilities were neutral, which was in line with some previous studies (e.g., McDougall et al., 2004; De Boer et al., 2012a). However, it is different from several studies in China, which found that peers held a negative attitude toward children with disabilities in inclusive settings (Ning, 2018; Zhang et al., 2018; Ma, 2019). The low acceptance of peers in this sample could be due to on the one hand that it investigated the attitude for all kinds of disabilities, while Ma's (2019) study focused on the attitude to children with ASD. Children with ASD are often less included in their classroom's social structure (Chamberlain et al., 2007; Rotheram-Fuller et al., 2010; Kasari et al., 2011). On the other hand, Ning (2018) and Zhang et al.'s (2018) studies surveyed preschool children and their attitude toward peers with disabilities, who may hold a more negative attitude than elementary students' since they rely more on the external behavior of the recipient and less on the reasons behind their behavior (Chen et al., 2012; Yu et al., 2012; Huckstadt and Shutts, 2014). Typically developing children may not reject children with disabilities because of the disabilities but instead, they may just not like children who are harmful or aggressive (Liu, 2015). Results also showed that girl students generally hold more positive attitude toward peers with disabilities. This result reflects those reported in previous research (e.g., De Boer et al., 2012b; Armstrong et al., 2017; Maggio et al., 2021). Specifically, in the comportments of attitude, the cognitive comportment referring to their psychological impression of peers with disabilities, including relevant facts, knowledge, and beliefs was the lowest. Ning (2018) found that physical inclusion can improve the positive affection and behavioral tendency of typical children toward peers with disabilities, while it does not promote recognition and understanding. It may result from the relationship among the three behavior level of attitude. Le (2006) states that there is a close relationship between the affective component and behavioral tendency component, whereas the cognitive component has higher independence.

Correlation analyses confirmed that there were positive correlations among inclusive climate, empathy, and attitude toward peers with disabilities. The positive association between inclusive climate and attitude toward peers with disabilities is consistent with previous studies by Horrocks (2006), Weller (2012), Gang et al. (2014). School climate is based on patterns of people's experiences of school life and reflects norms, goals, values, interpersonal relationships, teaching and learning practices, and organizational structures. The positive inclusive climate created in the general school environment, between teachers and students, and among students plays an important role in changing the negative–neutral attitude of the average student toward inclusive education (Cohen et al., 2009). The inclusive climate in the study is composed of teachers' support and students' emotional experience. On the one hand, successful implementation of any inclusive policy or programming is extremely dependent upon the educators being receptive and positive (Avramidis and Norwich, 2002). If students perceived teachers are caring, developing active programs, and providing individual attention to them, they are motivated to be more supportive and inclusive to others, including peers with disabilities (Potter, 2008). That is, they hold more positive attitude toward peers with disabilities. On the other hand, students who have positive emotional experiences with peers with disabilities may have developed a greater understanding of and sensitivity toward those peers and, therefore, perceives them more positively (McDougall et al., 2004).

We found that empathy was positively associated with typically developing students' attitude toward peers with disabilities. That is, students with a high level of empathy have higher acceptance of peers with disabilities in inclusive settings. This is in line with and strengthens further the results obtained by existing studies (e.g., Miller, 2013; Atkinson-Jones and Hewitt, 2019). The studies showed that the ability to enter into another individual's thoughts and feelings is important to develop in order to achieve a more inclusive attitude toward students with disabilities. Boele et al. (2019) consider empathy not only to develop high-quality relationships with peers but also to facilitate the quality of these relationships. Higher ability to share and understand others' emotions is related to more prosocial and less aggressive behavior toward others (Eisenberg et al., 2006) and better conflict resolution strategies (e.g., De Boer et al., 2012a). Students with high empathy are more likely to develop mutual trust, shared understanding, and optimal communication, allowing them to feel understood and listened to (Reynolds and Austin, 2000), which may contribute to their perception that the situation in inclusive settings for peers with disabilities situation is not fully appreciated. Ultimately, the typical students hold a more positive attitude toward peers with disabilities.

In order to verify whether the proposed interrelationships of inclusive climate, empathy, and attitude toward peers with disabilities hold true, the structural equation model statistical method was taken. The mediation analysis demonstrated that empathy was an intermediary factor affecting inclusive climate and typically developing students' attitude toward peers with disabilities. That means that typical students' attitude can be influenced by inclusive climate through empathy. When school systems are organized to include and meet the needs of all children from the local community, including those with a disability (Chen and Lu, 2006; Maggio et al., 2021), they are in the inclusive climate. In those schools, teachers are supportive and guide typical students to care and interact with peers with disabilities (Chen and Lu, 2006). Meanwhile, when students take the perspective of a peer in need, imagining how that peer is affected by his or her disabilities, empathetic feelings often occur (Panfile and Laible, 2012). Empathic feelings will be more generalized in schools with an inclusive climate. Once students feel empathy toward peers with disabilities, they induce the audience and improve their attitude toward their peers (Batson and Oleson, 1991).

## Limitations and further direction

Our study investigated the current status of peers' attitude to children with disabilities in inclusive settings and figured out the relationship between inclusive climate, empathy, and students' attitude. Regarding the investigation of peers' attitude toward students with disabilities, we applied a questionnaire including three attitude components. Some limitations need to be addressed. First, the sample of participants was not national, and the study was conducted in a developed city in China, which cannot represent the whole level of peers' attitude to children with disabilities in China. A national survey including different regions with different levels of economic development and different cultures on that research topic should be conducted in future studies. Second, considering the sociocultural characteristics of attitude toward individuals with disabilities, comparing them with students from other counties would be beneficial. Additional work should look closely at the context factors to provide more practical implications. Third, due to the primary students participating in the study knowing little about the category of disabilities, we did not analyze the influence of the category of disabilities of peers on the typically developing students. Further studies can explore the effect of the category of disabilities, which may provide more details on the implication of intervention to improve their attitude toward peers with different disabilities.

## Practical implications

First, the influence of the general school environment on the climate of inclusive education includes the support and attention to the smooth promotion of inclusive education under the leadership of the principal, the implicit influence of the campus culture, and the institutional protection at the school level. The support of principal provides both hard and soft support for the further development of inclusive education in regular schools, making it possible to develop a positive climate for inclusive education within the school and creating conditions for the formation of good relationships between students with special needs and their teachers and peers. Meanwhile, teachers should emphasize how to introduce children with disabilities to their typically developing classmates, and the information provided should promote a positive image of their peers with disabilities. Teachers can also take into account the work of emotional intelligence and self-regulation as fundamental strategies for improving the classroom climate.

Second, strategies should be taken to promote typically developing students' empathy. Behavioral strategies based on cooperative, equal-status, and personal contact can be applied to promote students' empathy toward peers with disabilities. Batson et al. (1997) found that once empathetic emotions are aroused, they are less vulnerable to information about victim responsibility. Once aroused, empathetic feelings appear to have some inertia and contribute to a positive attitude (Sharma et al., 2021). Teachers can use media and other visual materials to evoke empathy toward individuals with disabilities and guide them, forming positive attitude and sensitization among students of all ages toward disabled persons.

## Data availability statement

The datasets generated during and analyzed during the current study are available from the corresponding author on reasonable request.

## Ethics statement

The studies involving human participants were reviewed and approved by Beijing Normal University. Written informed consent to participate in this study was provided by the participants' legal guardian/next of kin.

## Author contributions

Conceptualization and funding acquisition: WF. Methodology and writing—original draft preparation: WF and YX. Data collection and analysis: CY and TZ. Writing—review and editing: WF and CY. All authors have read and agreed to the published version of the manuscript.
